# Model-Based ROC Curve: Examining the Effect of Case Mix and Model Calibration on the ROC Plot

**DOI:** 10.1177/0272989X211050909

**Published:** 2021-10-16

**Authors:** Mohsen Sadatsafavi, Paramita Saha-Chaudhuri, John Petkau

**Affiliations:** Faculty of Pharmaceutical Sciences, The University of British Columbia, Vancouver, BC, Canada; Faculty of Medicine, The University of British Columbia, Vancouver, BC, Canada; Department of Mathematics and Statistics, University of Vermont, Burlington, VT, USA; Department of Statistics, The University of British Columbia, Vancouver, BC, Canada

**Keywords:** clinical prediction models, model calibration, model validation, receiver-operating characteristic

## Abstract

**Background:**

The performance of risk prediction models is often characterized in terms of discrimination and calibration. The receiver-operating characteristic (ROC) curve is widely used for evaluating model discrimination. However, when comparing ROC curves across different samples, the effect of case mix makes the interpretation of discrepancies difficult. Further, compared with model discrimination, evaluating model calibration has not received the same level of attention. Current methods for examining model calibration require specification of smoothing or grouping factors.

**Methods:**

We introduce the “model-based” ROC curve (mROC) to assess model calibration and the effect of case mix during external validation. The mROC curve is the ROC curve that should be observed if the prediction model is calibrated in the external population. We show that calibration-in-the-large and the equivalence of mROC and ROC curves are together sufficient conditions for the model to be calibrated. Based on this, we propose a novel statistical test for calibration that, unlike current methods, does not require any subjective specification of smoothing or grouping factors.

**Results:**

Through a stylized example, we demonstrate how mROC separates the effect of case mix and model miscalibration when externally validating a risk prediction model. We present the results of simulation studies that confirm the properties of the new calibration test. A case study on predicting the risk of acute exacerbations of chronic obstructive pulmonary disease puts the developments in a practical context. R code for the implementation of this method is provided.

**Conclusion:**

mROC can easily be constructed and used to interpret the effect of case mix and calibration on the ROC plot. Given the popularity of ROC curves among applied investigators, this framework can further promote assessment of model calibration.

**Highlights:**

- Compared with examining model discrimination, examining model calibration has not received the same level of attention among investigators who develop or examine risk prediction models.
- This article introduces the model-based ROC (mROC) curve as the basis for graphical and statistical examination of model calibration on the ROC plot.
- This article introduces a formal statistical test based on mROC for examining model calibration that does not require arbitrary smoothing or grouping factors.
- Investigators who develop or validate risk prediction models can now also use the popular ROC plot for examining model calibration, as a critical but often neglected component in predictive analytics.

## Background

Risk prediction models that objectively quantify the probability of clinically important events based on observable characteristics are critical tools for efficient patient care. A risk prediction model is typically constructed using a development (or training) sample, but before it is adopted for use in a target population, its performance needs to be assessed in an independent (external) validation sample drawn from that population. In examining the appropriateness of a risk model, 2 fundamental aspects are discrimination and calibration. The former refers to the capacity of the model to properly stratify individuals with different risk profiles, and the latter refers to the degree to which predicted risks are close to the true risks.^1^

The receiver-operating characteristic (ROC) curve and the area under the ROC curve (AUC, or the c-statistic) are classical examples of tools for assessing model discrimination.^2^ When evaluating a risk prediction model in a sample, the discriminatory performance of the model can be affected by both the distribution of predictor variables (case mix) and the validity of the model in that sample.^3^ Consequently, when comparing the performance of a model between development and validation samples, differences in the case mix between the 2 samples can make comparisons difficult. One area of interest in the present work is to untangle these 2 sources of discrepancy. Early progress in this area was made by Vergouwe et al.^3^ who proposed benchmarks based on simulating responses from predicted risks and fitting the model in the validation sample. More recent work has largely focused on the AUC, an overall summary measure of the ROC curve.^4–6^

Compared with model discrimination, examining model calibration has not received the same level of attention.^7,8^ Model calibration is often neglected in the evaluation of the overall performance of risk prediction models, so much so that it is referred to as “the Achilles’ heel of predictive analytics.”^9^ In the context of a logistic model for binary responses, Van Calster et al.^10^ proposed a hierarchy of definitions for model calibration. In particular, a model is “moderately calibrated” if the average observed risk across all subjects with a given predicted risk is equal to the predicted risk. Moderate calibration is contrasted with mean calibration when the expected values of predicted and true risks are equal, with “weak” calibration when a linear calibration plot has an intercept of 0 and slope of 1, and with “strong” calibration when the predicted and observed risks are equal for all covariate patterns (an unrealistic condition in practical situations).^10^ Moderate calibration is typically assessed using the calibration plot, which shows the average value of the observed risk as a function of the predicted risk after grouping or smoothing response values.

In this work, we propose a model-based ROC (mROC) analysis. We show that the mROC connects ROC analysis, a classical means of evaluating model discrimination, to model calibration. With the help of a stylized example, we demonstrate how the mROC enables investigators to disentangle the effect of case mix and model validity on the shape of the ROC curve. We propose a novel statistical test for the assessment of model calibration that does not require specification of smoothing or grouping factors and evaluate its performance through simulation studies. Through a case study, we put the developments in a practical context.

## Notation and Context

Our main interest is in the “external validation” context, in which a previously developed risk prediction model for a binary outcome is applied to a new independent (external) sample to examine its performance in that sample’s target population. The risk prediction model is given by the deterministic function $\pi^{*}(X)$ , mapping an individual’s covariate vector X to $\pi^{*}$ , the probability of observing the binary outcome (response) of interest (e.g., whether a patient with asthma will experience a flare up in the next 6 mo). Let Y be the binary outcome of interest, with Y=1 indicating the presence of the disease or the occurrence of the event and 0 otherwise. In what follows, unless otherwise specified, by “calibration” we refer to moderate calibration (i.e., $P\left(Y=1|\pi^{*}(X)=p\right)=p$ ). Applying this model to the external sample consisting of a random sample of n individuals, we obtain $\pi^{*}=\left(\pi_{1}^{*},\ldots ,\pi_{n}^{*}\right)$ , the vector of predicted risks. In the external sample, we also observe the corresponding vector $Y=\;\left(Y_{1},\ldots ,Y_{n}\right)$ of response values.

## Empirical ROC Curve

Two fundamental probability distributions underlie the ROC curve: the distribution of predicted risks among individuals who experience the event (positive individuals, or cases) and among individuals who do not experience the event (negative individuals, or controls). Let $F_{1}$ and $F_{0}$ represent the corresponding cumulative distribution functions (CDFs) of the predicted risk:

$$\begin{array}{c} F_{1}(t)=P(\pi^{*}\leq t|Y=1),\\ F_{0}(t)=P(\pi^{*}\leq t|Y=0). \end{array}$$

The true-positive and false-positive probabilities are closely linked with the distribution of risk among the positive and negative individuals, respectively: $\mathrm{TP}\left(t\right)\equiv P\left(\pi^{*}>t|Y=1\right)=1-F_{1}\left(t\right)$ , and $\mathrm{FP}\left(t\right)\equiv P\left(\pi^{*}>t|Y=0\right)=1-F_{0}\left(t\right)$ . The population ROC curve induced by the risk prediction model $\pi^{*}$ can be expressed as

$$\mathrm{ROC}\left(t\right)=1-F_{1}(F_{0}^{-1}(1-t)),$$

where 0≤t≤1 is the false-positive probability.^11^

With the external data set, consistent estimators for $F_{1}$ and $F_{0}$ can be obtained by averaging the indicators $I\left(\pi_{i}^{*}\leq t\right)$ for each of the positive and negative groups:

$$F_{1n}\left(t\right)=\frac{\sum_{i=1}^{n}\{I\left(\pi_{i}^{*}\leq t\right)Y_{i}\}}{\sum_{i=1}^{n}Y_{i}},$$

and

$$F_{0n}\left(t\right)=\frac{\sum_{i=1}^{n}\{I\left(\pi_{i}^{*}\leq t\;\right)(1-Y_{i})\}}{n-\sum_{i=1}^{n}Y_{i}}.$$

$F_{1n}\left(t\right)$ and $F_{0n}\left(t\right)$ are used to generate $\mathrm{RO}C_{n}\left(t\right)$ , the empirical ROC, as a consistent estimator of the population ROC curve.^12,13^

## mROC Curve

The *i*^th^ subject in the external sample is a random draw from the set of all individuals in the target population whose predicted risk is $\pi_{i}^{*}$ . Hence, under the assumption that the model is calibrated, we have $P(Y_{i}=1)=P(Y=1|\pi^{*}(X)=\pi_{i}^{*})=\pi_{i}^{*}$ ; that is, the vector of observed response values is a random draw of independent Bernoulli trials from the vector of predicted risks. Hence, in addition to the ROC curve based on the observed responses, one can construct an ROC curve based on the potential random responses generated from the Bernoulli distribution with probabilities equal to the predicted risk.

Let $Y^{*}$ be a random realization of this potential response from the predicted risk of a randomly selected individual. The ROC-related CDFs based on $Y^{*}$ are

$${\bar{F}}_{1}\left(t\right)=P\left(\pi^{*}\leq t|Y^{*}=1\right),$$

and

$${\bar{F}}_{0}\left(t\right)=P\left(\pi^{*}\leq t|Y^{*}=0\right).$$

The application of Bayes’s rule leads to the following estimators in the external sample:

$${\bar{F}}_{1n}\left(t\right)=\frac{\sum_{i=1}^{n}I\left(\pi_{i}^{*}\leq t\;\right)\pi_{i}^{*}}{\sum_{i=1}^{n}\pi_{i}^{*}},$$

and

$${\bar{F}}_{0n}\left(t\right)=\frac{\sum_{i=1}^{n}I\left(\pi_{i}^{*}\leq t\;\right)(1-\pi_{i}^{*})}{n-\sum_{i=1}^{n}\pi_{i}^{*}}.$$

Hence, one can generate a “model-based” ROC or $\mathrm{mRO}C_{n}\left(t\right)$ , independently of the observed outcomes in the external sample, based on the CDFs ${\bar{F}}_{1n}$ and ${\bar{F}}_{0n}$ obtained by averaging the indicator functions $I\left(\pi_{i}^{*}\leq t\;\right)$ with weights of $\pi_{i}^{*}/\sum^{\text{​}}\pi_{i}^{*}$ and $(1-\pi_{i}^{*})/\sum^{\text{​}}(1-\pi_{i}^{*})\;$ for the *i*^th^ individual in the sample. This is an extension of the definition of the model-based c-statistic proposed by van Klaveren et al.^5^ to the entire ROC curve. As demonstrated in Supplementary Material section 1, the area under the mROC curve is equal to the model-based c-statistic.^5^

## Connection Between the mROC Curve, Case Mix, and Model Calibration

The limiting forms (population equations) of the estimated CDFs $F_{1n}$ , $F_{0n}$ , ${\bar{F}}_{1n}$ , and ${\bar{F}}_{0n}$ are derived in Supplementary Material section 2. An important consequence is that, provided that the model is calibrated in the external sample, $\mathrm{RO}C_{n}\left(t\right)$ and $\mathrm{mRO}C_{n}\left(t\right)$ converge to the same value at each point t , as n , the sample size in the external sample, approaches infinity. That is, moderate calibration is a sufficient condition for the convergence of empirical ROC and mROC curves. A stylized example demonstrating how mROC is affected by model miscalibration is provided in Supplementary Material section 3.

Unlike in the expression of $F_{1n}$ and $F_{0n}$ , the observed outcomes in the sample do not appear in the expression of ${\bar{F}}_{1n}$ and ${\bar{F}}_{0n}.$ The behavior of these CDFs depends on the predicted risks, rather than the observed outcomes in the sample. Therefore, the mROC curve depicts the case-mix–adjusted ROC curve—the ROC curve that would be expected to be observed in the sample, if the model is calibrated in this sample. This motivates our proposal for using mROC to gain insight into the effect of case mix and model calibration when examining the external validity of a model.

**Figure 1:**
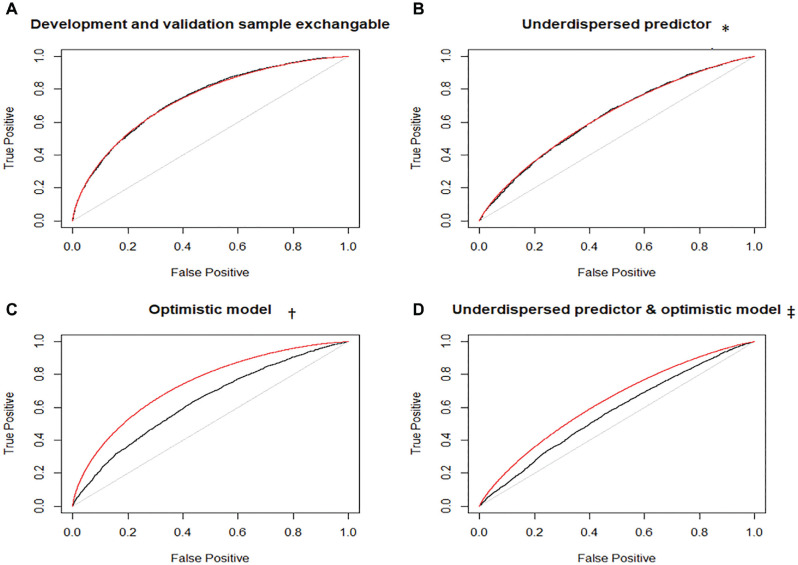
Empirical receiver-operating characteristic (ROC; black) and model-based ROC (mROC; red) curves for the stylized example. *Distribution of the single predictor in the validation population: X∼Normal(µ= 0, σ = 0.5). ^†^Association model in the validation population: P(Y = 1) = 1/(1 + exp(−X/2)). ^‡^Predictor distribution same as in panel B, and association model same as in panel C.

Consider the mROC and empirical ROC curves in the validation sample when examining the external validity of a model. The former carries the association between the predictors and outcome from the development sample through the prediction model, whereas the latter captures such association in the validation sample. However, both are based on the case mix in the validation sample. Because of the shared case mix, discrepancies between these curves point toward model miscalibration in the validation sample. This can be demonstrated using a stylized example: Consider a single predictor X , which has a standard normal distribution in the development population. Using a sample from the development population, we construct a risk prediction model as P(Y=1)=1/(1+exp(−X)) , which happens to be the correctly specified model (and thus is calibrated) in this population. This model has an AUC of 0.740 in the development population. Now consider 4 hypothetical external validation scenarios. In the first scenario (Figure 1A), the distribution of X and its association with the outcome are the same in the validation population as in the development population. As such, the empirical ROC and mROC curves agree (and will also resemble the empirical ROC curve in the development sample). In the second scenario (Figure 1B), the predictor is underdispersed in the validation population (SD = 0.5), while the association is still the same; thus, the model is calibrated. Given the lower variance of the risks, the model has lower discriminatory power in this population (AUC = 0.641). Both the empirical ROC and mROC curves move closer to the diagonal line, but they closely match each other. Next, consider a validation population that has the same distribution of X as the development population, but with a weaker predictor-outcome association ( P(Y=1)=1/(1+exp(−X/2)) ; thus, the model is “optimistic” and not calibrated. This again causes the empirical ROC curve to be closer to the diagonal line (Figure 1C, AUC = 0.641). However, the mROC curve remains unchanged from the first scenario. This pattern indicates that the change in the discriminatory performance of the model between the development and validation samples is due to model miscalibration in the validation sample. Finally, consider a validation population in which the predictor is underdispersed and the association is weaker (Figure 1D). Both factors contribute to the empirical ROC curve being closer to the diagonal line (AUC = 0.584). Here, because of the difference in the case mix, the mROC curve also gets closer to the diagonal line, but because of the miscalibrated model in the validation sample, it is not aligned with the empirical ROC curve. This demonstration implies that difference in case mix between the development and validation samples does not lead to the discrepancy between the mROC curve and the empirical ROC curve; however, miscalibration of the prediction model in the external sample can lead to discrepancy.

## mROC as the Basis of a Novel Statistical Test for Model Calibration

Although moderate calibration is a sufficient condition for the convergence at all points of the empirical ROC and mROC curves, moderate calibration on its own might not be a necessary condition for such convergence. To progress, in Supplementary Material section 4 we show that at the population level, the equivalence of ROC and mROC curves guarantees moderate calibration if an additional condition is imposed. This condition is mean calibration, i.e., $E(\pi^{*})=E(Y)$ , a condition whose assessment is an integral part of external validation of a risk prediction model.^14^

Based on this finding, we propose a statistical inference procedure. We define the null hypothesis ( $H_{0}$ ) as the model being calibrated: $P(Y=1|\pi^{*}=p)=p$ . Given the results presented in the Supplementary Material section 4, $H_{0}$ can be seen as a combination of 2 null hypotheses, one on the equivalence of the expected values of predicted and observed risks ( $H_{0A}$ ) and the other on the equivalence of the mROC and ROC curves ( $H_{0B}$ ):

$$H_{0}:\left\{ \begin{array}{cc} \begin{array}{c} H_{0A} \end{array} & E\left(\pi^{*}\right)=E(Y)\;\mathrm{mean}\;\mathrm{calibration}\\ H_{0B} & \forall t\;\mathrm{mROC}\left(t\right)=\mathrm{ROC}\left(t\right)\;\mathrm{mROC}/\mathrm{ROC}\mathrm{equality} \end{array}\right.$$

These hypotheses jointly provide the necessary and sufficient conditions for the risk prediction model to be calibrated.

For $H_{0A}$ , consider $A=|E\left(Y\right)-E\left(\pi^{*}\right)|$ . This population quantity achieves its minimum value of 0 if $H_{0A}$ is true. Our proposed test statistic is the sample estimator of this quantity, the absolute average distance between the observed and predicted risks in the sample:

$$A_{n}=\frac{1}{n}|\sum_{i=1}^{n}(Y_{i}-\pi_{i}^{*})|\;\left(\mathrm{mean}\;\mathrm{calibration}\;\mathrm{statistic}\right).$$

For $H_{0B}$ , consider the population quantity $B=\int_{0}^{1}\left|\mathrm{ROC}\left(t\right)-\mathrm{mROC}\left(t\right)\right|\mathrm{dt}$ , which achieves its minimum value of 0 when the ROC and mROC curves are equal at all points. Our proposed test statistic is a sample estimator for this quantity, the integrated absolute difference between the empirical ROC and mROC curves in the sample:

$$B_{n}=\int_{0}^{1}\left|\mathrm{RO}C_{n}\left(t\right)-\mathrm{mRO}C_{n}\left(t\right)\right|\mathrm{dt}\;\left(\mathrm{mROC}/\mathrm{ROC}\;\mathrm{equality}\;\mathrm{statistic}\right).$$

Given that both $\mathrm{RO}C_{n}$ and $\mathrm{mRO}C_{n}$ are step functions, the above integral is the sum of rectangular areas and can be evaluated exactly.

The null distributions of both $A_{n}$ and $B_{n}$ can be approximated numerically through straightforward Monte Carlo simulations. Through simulating vectors of response values from the vector of predicted probabilities, one can generate many simulated ROC curves and use them to construct empirical distribution functions under $H_{0}$ for $A_{n}$ and $B_{n}$ . These empirical distributions can then be used to generate approximate one-tailed *P* values for these 2 statistics as

$$p_{A_{n}}=1-\mathrm{eCD}F_{A_{n}}\left(A_{n}\right),$$

where $\mathrm{eCD}F_{A_{n}}$ is the empirical CDF of the mean calibration statistic under $H_{0},$ and

$$p_{B_{n}}=1-\mathrm{eCD}F_{B_{n}}\left(B_{n}\right),$$

where $\mathrm{eCD}F_{B_{n}}$ is the empirical CDF of the mROC/ROC equality statistic under $H_{0}$ .

Individually, the 2 statistics provide insight about the performance of the model. However, it is more desirable to obtain a single overall *P* value for $H_{0}$ . If these tests were independent, one could use Fisher’s method^15^ to obtain a unified *P* value, as under $H_{0}$ , $p_{A_{n}}$ and $p_{B_{n}}$ have standard uniform distributions. Thus, the statistic

$$U_{n}=-2\left[\log\left(p_{A_{n}}\right)+\log\left(p_{B_{n}}\right)\right]$$

would have a chi-square distribution with 4 degrees of freedom. However, as the 2 statistics are generated from the same data, they are dependent. An adaptation of Fisher’s method for dependent *P* values (based on matching the moments of the test statistic to that of a chi-square distribution) can be used.^16^ The steps for generating a unified *P* value are outlined in the algorithm provided in Supplementary Material section 5.

## Simulation Studies

We performed simulation studies to evaluate the finite-sample properties of the proposed test and compare its performance against the conventional Hosmer-Lemeshow and likelihood ratio tests of model calibration. We modeled a single predictor X with a standard normal distribution and the true risk as p=1/(1+exp(−X)) . We evaluated the performance of the tests in a simulated independent sample of n observations when the predicted risks suffer from various degrees of miscalibration. Two sets of simulations were performed. In the first set, we assumed the risk model generated potentially miscalibrated predictions in the form of $logit\left(\pi^{*}\right)=a+b.logit\left(p\right)=a+b.X.\;$ Given the linear association on the logit scale between the predicted and actual risks, weak and moderate calibration are equivalent in these scenarios. Therefore, the likelihood ratio test (simultaneously testing whether a=0 and b=1 ) has the maximum theoretical power in detecting miscalibration. As such, this simple setup provides an opportunity to judge the performance of the unified test against a gold standard.

**Figure 2:**
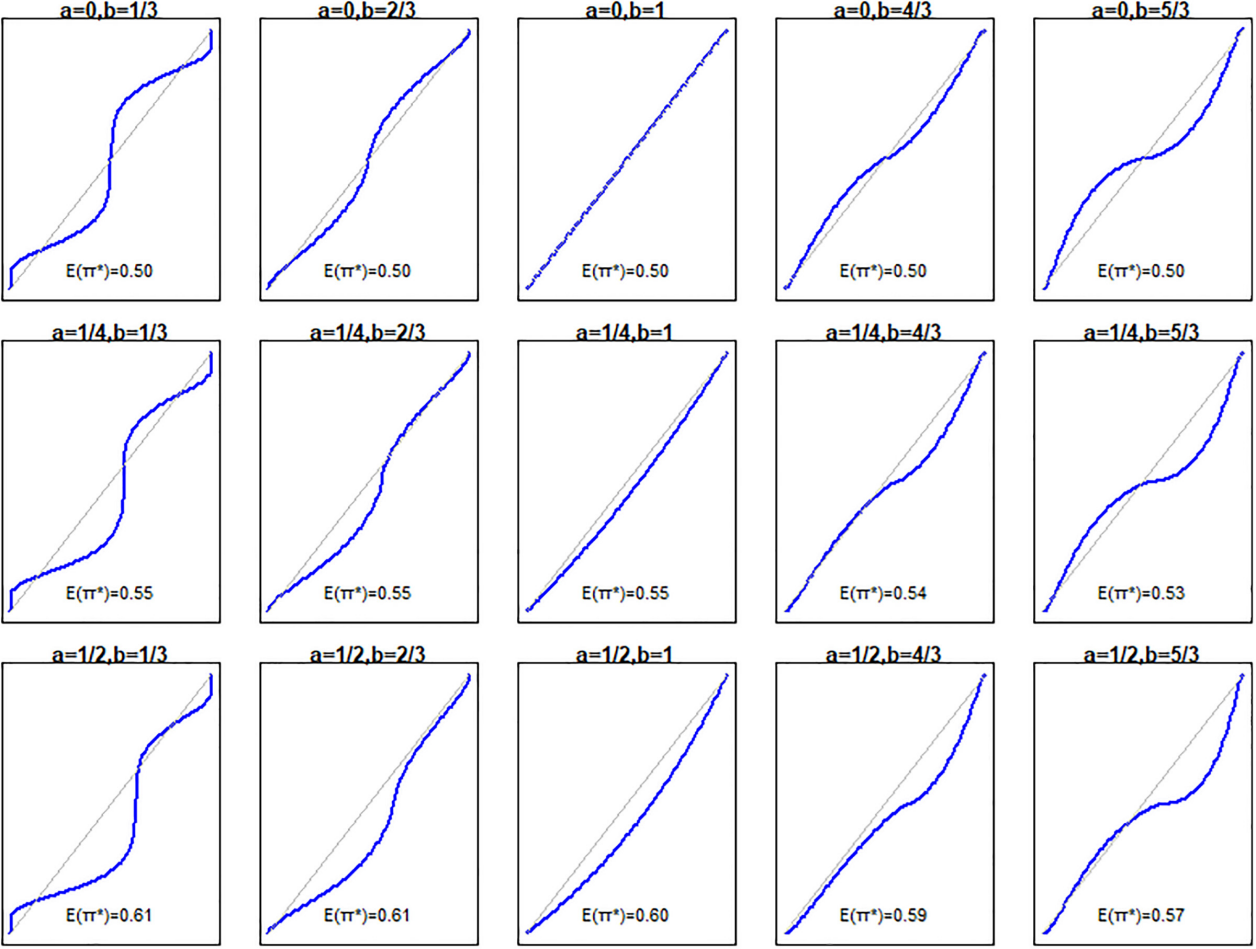
Relationship between predicted (*x* axis) and true (*y* axis) risks for the simulation scenarios.

In the second set, the true risk model remained the same as above, and we modeled nonlinear miscalibrations as $\mathrm{logit}\left(\pi^{*}\right)=a+b.\mathrm{sign}\left(X\right).{\left|X\right|}^{1/b}$ . Here, a affects the mean calibration, whereas the term involving b is an odd function that flexibly changes the calibration slope but preserves the expected value of the predicted risks. We simulated response values and predicted risks with values $a=\left\{0,\frac{1}{4},\frac{1}{2}\right\}$ and $b=\left\{\frac{1}{3},\frac{2}{3},1,\frac{4}{3},\frac{5}{3}\right\}$ , with 3 different sample sizes: n={100,250,1000} , in a fully factorial design (45 scenarios). Figure 2 presents the population-level calibration plots for each combination of a and b and the average predicted risks under each transformation.

We calculated the power of the mean calibration test, the mROC/ROC equality test, the unified test, the Hosmer-Lemeshow test (based on decile groups), and the likelihood ratio test in detecting miscalibration at the 0.05 significance level. Following recommendations on objectively deciding on the number of simulations,^17^ we obtained the results through 2500 Monte Carlo iterations such that the maximum SE around the probability of rejecting H_0_ would be 0.01. Within each iteration, *P* values were calculated from $\;\mathrm{eCD}F_{A_{n}}$ and $\mathrm{eCD}F_{B_{n}}$ that were in turn based on 10^5^ simulations. We used R for this analysis,^18^ with the implementation of the simulation-based estimation of $\mathrm{eCD}F_{A_{n}}$ and $\mathrm{eCD}F_{B_{n}}$ in C for computational efficiency.

**Figure 3:**
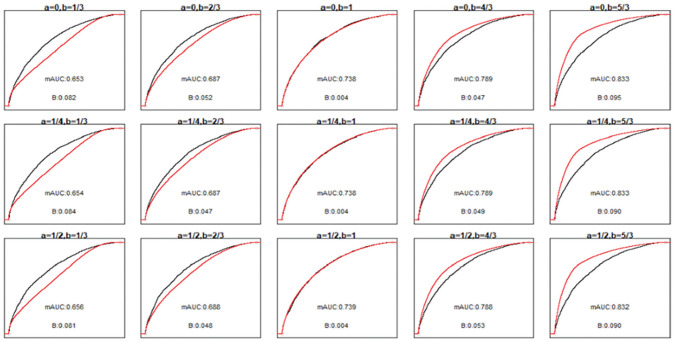
Receiver-operating characteristic (ROC; black) and model-based ROC (mROC; red) curves for the second simulation scenario. The panels positionally correspond to the calibration plots and simulation parameters presented in Figure 2. The ROC curves approximate the population-level curves as they are based on a large sample size (10,000 simulated observations). The area under the ROC curve is 0.740 in all scenarios. ROC, receiver operating characteristic; B, ROC equality statistic; mAUC, area under the model-based ROC curve.

Results of the first set of simulations are provided in Supplementary Material section 6. The power of the unified test was very close to that of the likelihood ratio test across all scenarios examined. Figure 3 provides the empirical ROC and mROC curves for the second set of simulations. As all the mappings from p to $\pi^{*}$ in these simulations are monotonic, the ROC curve remains the same in all panels (with an AUC of 0.740). However, the mROC is generally affected by miscalibration.

**Figure 4:**
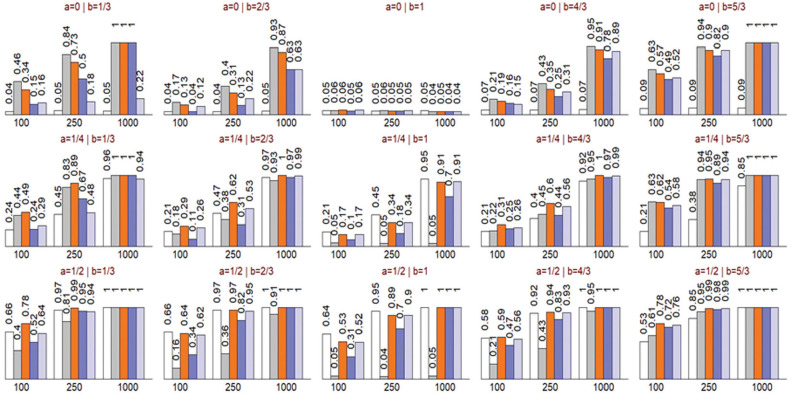
Probability of rejecting the null hypothesis for the mean calibration (white bar), receiver-operating characteristic (ROC) equality (gray bar), unified (orange bar), Hosmer-Lemeshow (dark blue bar), and likelihood ratio (light blue bar) tests. The panels positionally correspond to the calibration plots and simulation parameters presented in Figure 2. Results are based on 2,500 simulations for each scenario.

The performances of all tests are summarized in Figure 4. The middle panel on the top row, where a=0 and b=1 , pertains to the only scenario where H_0_ is true. All tests appropriately rejected the null hypothesis around the nominal type I error rate of 0.05. Focusing on the first row, given a=0 , $E\left(\pi^{*}\right)=E(Y)=0.5$ under these transformations; thus, $A_{n}$ (mean calibration, the white bars) does not detect the miscalibration ( $p_{A_{n}}$ remains ∼0.05). On the other hand, in the third column, where b=1 , and thus the predicted odds are proportional to the true odds, the mROC and ROC curves remain very close to each other, and $B_{n}$ does not detect the miscalibration under such transformations ( $p_{B_{n}}$ remains ~0.05; Figure 3). However, in all scenarios where miscalibration was present, the unified test rejected the null hypothesis with $p_{U_{n}}>0.05$ . In general, the power of the unified test was either equal to or higher than that of the Hosmer-Lemeshow and likelihood ratio tests. The latter, being a test of weak calibration, can have low power when the miscalibration is S-shaped such that the calibration slope remains unchanged (e.g., in the top left panel when *a* = 0 and *b* = 1/3, with 22% power with a sample size of 1000, compared with >99% power for the unified and Hosmer-Lemeshow tests).

## Application

Chronic obstructive pulmonary disease (COPD) is a common chronic disease of the airways. Periods of intensified disease activity, referred to as exacerbations, are an important feature of the disease. Individuals vary widely in their tendency to exacerbate.^19^ Predicting who is likely to experience an exacerbation, especially a severe one that will require hospital admission, will provide opportunities for preventive interventions.^20^

We used data from the MACRO^21^ and STATCOPE,^22^ two clinical trials in COPD patients with exacerbations as the primary outcome, to, respectively, develop and validate a risk prediction model for the occurrence of COPD exacerbations in the first 6 mo of follow-up. Baseline characteristics of both samples are provided in Table 1.

**Table 1:** Baseline Characteristics and Outcomes for MACRO and STATCOPE Samples

Sample Characteristics	Development Sample (MACRO)	Validation Sample (STATCOPE)	
Sample size	1,074	832	
Number (%) with at least 1 exacerbation during the first 6 mo of follow-up			
All exacerbations	691 (64.3%)	454 (54.5%)	
Severe exacerbations	141 (13.1%)	73 (8.8%)	
Female sex (%)	59.2	56.6
Age (y), mean (IQR)	65.2 (13.0)	62.4 (13.0)
Previous history of oxygen therapy (%)	59.3	48.4
Previous history of hospitalization (%)	50.0	31.1
SGRQ, mean (IQR)	50.1 (22.4)	49.6 (24.4)
FEV_1_ (L), mean (IQR)	1.11 (0.70)	1.19 (0.81)
Current smoker (%)	21.7	29.7
Current LABA user (%)	74.4	42.6
Current LAMA user (%)	63.5	66.1

IQR, interquartile range; SGRQ, St. George Respiratory Questionnaire; FEV_1_, forced expiratory volume at 1 s; LABA, long-acting beta agonist; LAMA, long-acting antimuscarinic agent.

Of note, these data have previously been used for a more sophisticated prediction model.^23^ Here, we focus on a simpler approach as the nuances of model development are beyond the scope of this work. We used a logistic regression model based on the data from the MACRO trial that included the predictors as listed in Table 1 based on an a priori list of covariates generated from prior knowledge of possible association with the outcome. We considered 2 outcomes: all exacerbations and severe exacerbations, and we developed 2 distinct models. The regression coefficients for both models are provided in Table 2. The study was approved by the University of British Columbia and Providence Health Research Ethics Board (H11–00786).

**Table 2:** Regression Coefficients for the Risk Prediction Models (Based on the MACRO Sample) for All and Severe Exacerbations

Log-Odds Ratio^a^	All Exacerbations, Estimate (SE)	Severe Exacerbations, Estimate (SE)
Intercept	0.787 (0.707)	–3.840 (1.018)
Female sex	–0.482 (0.145)	0.209 (0.201)
Age (/10)^b^	–0.094 (0.084)	–0.016 (0.119)
Previous history of oxygen therapy	0.275 (0.147)	0.297 (0.217)
Previous history of hospitalization	0.490 (0.135)	0.925 (0.200)
SGRQ^b^	0.098 (0.043)	0.219 (0.063)
FEV_1_ (L)^b^	–0.158 (0.146)	–0.251 (0.219)
Current smoker	–0.168 (0.176)	–0.017 (0.242)
Current LABA user	0.157 (0.155)	0.466 (0.247)
Current LAMA user	0.354 (0.142)	0.083 (0.206)

SE, standard error; SGRQ, St. George Respiratory Questionnaire; FEV_1_, forced expiratory volume at 1 s; LABA, long-acting beta agonist; LAMA, long-acting anti-muscarinic agent.

a We included a coefficient for randomized treatment (azithromycin), but it was set to 0 for prediction (as the model is applicable to those who are not on preventive therapy, and none of the individuals in the validation sample were on such a therapy).

b Log-odds ratios are for a 1-unit increase for continuous variables

**Figure 5:**
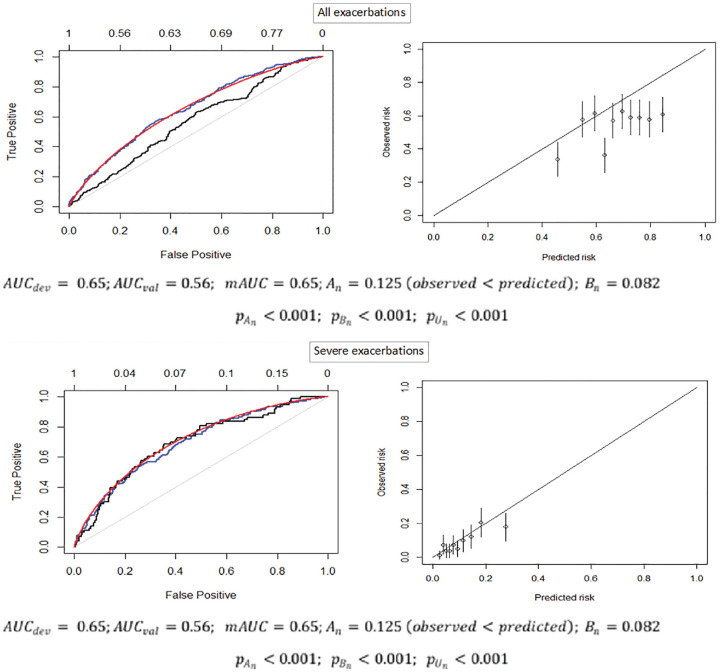
The empirical ROC curves from the MACRO development (blue) and STATCOPE validation (black) samples, the mROC curve from the STATCOPE validation sample (red; left panels), and the calibration plot (right panels). AUC_dev_, area under the curve (c-statistic) in the development sample; AUC_val_, area under the curve (c-statistic) in the validation sample; mAUC, area under the model-based ROC curve A_n_, mean calibration statistic; B_n_, ROC equality statistic; pA_n_): *p* value of the mean calibration test; p(B_n_), *p* value for the ROC equality test; p(U_n_), *p* value of the unified test.

Figure 5 provides the empirical ROC curve from the development sample (MACRO) as well as the empirical ROC and mROC curves from the validation sample (STATCOPE) and the calibration plot for both outcomes. For all exacerbations, the mROC curve was very close to the development empirical ROC curve but not to the external empirical ROC curve. This indicates that the reduction in the discriminatory performance of the model in the validation sample is due to miscalibration. Indeed, both components of the proposed test indicated a departure from calibration. The mean calibration test produced $p_{A_{n}}<0.001$ (a 2-tailed *t* test also had a *P* value <0.001). This was also the case for the equivalence of the mROC and empirical ROC curves in the validation sample ( $p_{B_{n}}<0.001$ ). The unified test also rejected the hypothesis that the model is calibrated ( $p_{U_{n}}<0.001$ ). As well, the Hosmer-Lemeshow test produced a *P* value of <0.001. The calibration plot in the external sample suggested miscalibration, with a general overestimation of risk.

The model for severe exacerbations had higher discriminatory power. All 3 ROC curves were generally aligned with each other. The mean calibration test produced $p_{A_{n}}=0.070$ (a 2-tailed *t* test led to p=0.061 ), whereas the mROC/ROC equality test resulted in $p_{B_{n}}=0.74$ . The unified test did not indicate evidence against moderate calibration ( $p_{U_{n}}=0.20$ ). The Hosmer-Lemeshow test resulted in a *p* value of 0.16. The calibration plot suggested generally good agreement between the predicted and observed risks for all but the highest decile of predicted risk (Figure 5).

## Discussion

This article provides an introduction of the model-based ROC (mROC) curve, the ROC curve that should be expected if the model is at least moderately calibrated in an external validation sample. We showed that moderate calibration is a sufficient condition for the convergence of empirical ROC and mROC curves. We extended these results by proving that, together, the mean calibration and the equivalence of mROC and ROC curves in the population are sufficient conditions for the model to be moderately calibrated. To test for such equivalences within a sample, we suggested a simulation-based test. Our simulations empirically verified the postulated properties of this novel test. To the best of our knowledge, this is the first time that the ROC plot, a classical means of communicating model discrimination, has been connected to model calibration. We have implemented the proposed methodology in an R package, which is available from https://github.com/resplab/predtools/.

Previous investigators have suggested using case mix–corrected performance metrics in judging the external validation of a model. Vergouwe et al.^3^ proposed a general approach for calculating different model-based metrics by simulating responses from predicted risks in the validation sample and comparing the resulting metrics with the empirical ones in the validation sample. Van Klaveren et al^5^ focused on one such metric, the c-statistic, and developed closed-form estimators that would quantify the expected change in a model’s discriminative ability due to case-mix heterogeneity. Our methodology extends such work to the entire ROC curve and in doing so establishes a connection between mROC/ROC equality and model calibration that enables formal statistical inference on moderate calibration. The test that is classically associated with calibration plots is the Hosmer-Lemeshow test, which is criticized because of its sensitivity to the grouping of the data and lack of information about direction of miscalibration.^24^ Our proposed test is free from arbitrary grouping of the data or the choice of smoothing factors. Given the shortcomings of the Hosmer-Lemeshow test, alternative inferential techniques for evaluating model calibration have been proposed. Allison^24^ reviewed the measures of fit of logistic regression models and categorized them as indices of predictive power (such as *R*^2^) and goodness of fit. In their comprehensive review of goodness-of-fit tests for logistic models,^25^ Hosmer et al. defined goodness-of-fit as the adequacy of a model on 3 fronts: the link function, the probability distribution, and the linear predictor. This is a distinctly different pursuit than examining moderate calibration. Consequently, none of the tests examined by Allison and Hosmer et al. can be considered a test for moderate calibration. Our proposed test seems to be the first alternative to the Hosmer-Lemeshow test that strictly examines moderate calibration.

These developments can be used in practice in different ways. Steyerberg and Vergouwe^14^ have proposed an “ABCD” approach for external validation of a model (where A is the mean calibration; B, calibration slope; C, c-statistic; and D, decision curve analysis).^14^ The B step in their approach can be replaced with the mROC’s B statistic, which, together with the A step (which is the same as the A step in the unified test), will test moderate calibration, the most desired form of calibration, as opposed to weak calibration tested via calibration slope.^10^ Further, if the research involves simultaneous model development and external validation, drawing the empirical ROC curves from both samples alongside the mROC curve in the validation sample will provide visual information on the causes of difference in the performance of the model between the 2 samples (as demonstrated in our case study). Incompatibility between mROC and empirical ROC in the validation sample will rule out moderate calibration. Conversely, while agreement between the 2 curves does not rule in moderate calibration per se, it does so provided that mean calibration (calibration-in-the-large) is achieved. This visual interpretation can be augmented with formal hypothesis testing using the proposed unified statistic. Such comparisons can also be made for subgroups within the sample, although multiple hypothesis testing should be controlled for in such circumstances. Even when the investigators are not planning to produce ROC curves, the proposed test for moderate calibration can be reported independently. This can complement the scalar metrics that measure the degree of miscalibration but are not based on formal hypothesis testing, such as Harrell’s Emax,^26^ Austin and Steyerberg’s Integrated Calibration Index,^27^ and Van Hoorde et al.’s Estimated Calibration Index.^28^

There are several ways the proposed methodology can be extended. The ROC curve has been extended to categorical^29^ as well as to time-to-event data,^30,31^ and similar developments can also be pursued for the mROC methodology. Development of inferential methods that would not require Monte Carlo simulations can also be of potential value. As the ROC curve can be interpreted as a CDF,^11^ nonparametric statistics based on the distance between CDFs can conceivably be developed to test the equivalence of mROC and ROC curves. However, the calculation of the simulation-based *P* value for the ROC equality test is computationally efficient (except for very large data sets). Thus, Monte Carlo error can be made smaller than the error generated from applying asymptotic methods to a finite sample. Further, although we have shown that mROC/ROC compatibility per se does not guarantee model calibration, our simulations suggest that such compatibility occurs when predicted and calibrated risks are proportional on the odds scale. As such, mROC/ROC compatibility might mean one should adjust the intercept term in a logistic regression model only to achieve moderate calibration. In this sense, our proposed approach has some similarities with the stepwise approach proposed by Vergouwe et al.^32^ for examining which aspect of a risk prediction model (mean calibration, calibration slope, or individual regression coefficients) needs to be updated to improve the performance of the model in a new sample. However, our simulations were proof of concept, and this observation should be further corroborated by theoretical developments or more extensive simulations.

One of the promises of precision medicine is to empower patients in making informed decisions based on their specific risk of outcomes.^33^ Basing medical decisions on miscalibrated predictions can be harmful. Our contribution is the development of mROC analysis, a simple method for separating the effect of case mix and model miscalibration on the ROC curve and for inference on model calibration. Recent arguments and counterarguments indicate that the methodological research community is divided in its opinion on the utility of ROC curves in the assessment of risk prediction models.^34,35^ ROC curves, however, remain a widely adopted tool among applied researchers in understanding and communicating the discriminatory performance of such models. The mROC methodology adds to the utility of ROC curves by enabling the examination of model calibration using the ROC plot. These developments can result in more attention to model calibration as an often-neglected but crucial aspect in the development of risk prediction models.

## Supplemental Material

sj-docx-1-mdm-10.1177_0272989X211050909 – Supplemental material for Model-Based ROC Curve: Examining the Effect of Case Mix and Model Calibration on the ROC Plot Supplemental material, sj-docx-1-mdm-10.1177_0272989X211050909 for Model-Based ROC Curve: Examining the Effect of Case Mix and Model Calibration on the ROC Plot by Mohsen Sadatsafavi, Paramita Saha-Chaudhuri and John Petkau in Medical Decision Making
