# Supplementary material for: Model-Based ROC Curve: Examining the Effect of Case Mix and Model Calibration on the ROC Plot
Source: Med Decis Making. 2021 Oct 16;42(4):487–99. doi: 10.1177/0272989X211050909 (PMC9005838; doi:10.1177/0272989X211050909)
Supplement: sj-docx-1-mdm-10.1177_0272989X211050909 – Supplemental material for Model-Based ROC Curve: Examining the Effect of Case Mix and Model Calibration on the ROC Plot [file sj-docx-1-mdm-10.1177_0272989X211050909.docx]

Supplementary Material for

Model-based ROC (mROC) curve: examining the effect of case-mix and model calibration on the ROC plot

Mohsen Sadatsafavi; Paramita Saha-Chaudhuri; John Petkau

**Table of content**

Section 1: Proof of the equivalence of area under mROC and model-based c-statistic

Section 2: Proof of the convergence of mROC and empirical ROC curves under model calibration

Section 3: A stylized example demonstrating the connection between mROC and model ­­­­­calibration

Section 4: Sufficient conditions for moderate calibration

Section 5: Calculating a unified p-value for the assessment of model calibration

Section 6: Results of the first set of simulations

**Section 1:** Proof of the equivalence of area under mROC and model-based c-statistic

Proposition: Let $f(.)$ be the probability density function of the random variable X. Let $G(.)$ be the cumulative distribution function of an independent continuous random variable Y. If both X and Y take values in [0,1], then

$$P\left( X>Y \right)=\int_{0}^{1} P\left( X>Y|X=z \right)dP\left( X\leq z \right)=\int_{0}^{1} P\left( Y<z \right)f\left( z \right)dz=\int_{0}^{1} f\left( z \right)G\left( z \right)dz$$

Proof of the main claim: As in the main text, let Y* be a model-based response, and define $\bar{F}_{0}$ and $\bar{F}_{1}$ as the CDFs underlying mROC (the CDFs of predicted risks among individuals with Y* of 0 and 1, respectively). Let $\pi_{0}^{*}$ and $\pi_{1}^{*}$ be random draws from these two distributions . The model-based c-statistic (mbc) is the probability that among two individuals with discordant model-based responses, that with $Y^{*}=1$ has a higher predicted risk than that with $Y^{*}=0$. That is,$mbc=P\left( \pi_{1}^{*}>\pi_{0}^{*} \right) ADDIN ZOTERO\_ITEM CSL\_CITATION \{"citationID":"eMUV2wT5","properties":\{"formattedCitation":"(2)","plainCitation":"(2)","noteIndex":0\},"citationItems":[\{"id":35,"uris":["http://zotero.org/users/65991/items/9WYCX2UF"],"uri":["http://zotero.org/users/65991/items/9WYCX2UF"],"itemData":\{"id":35,"type":"article-journal","abstract":"Concordance measures are frequently used for assessing the discriminative ability of risk prediction models. The interpretation of estimated concordance at external validation is difficult if the case-mix differs from the model development setting. We aimed to develop a concordance measure that provides insight into the influence of case-mix heterogeneity and is robust to censoring of time-to-event data. We first derived a model-based concordance (mbc) measure that allows for quantification of the influence of case-mix heterogeneity on discriminative ability of proportional hazards and logistic regression models. This mbc can also be calculated including a regression slope that calibrates the predictions at external validation (c-mbc), hence assessing the influence of overall regression coefficient validity on discriminative ability. We derived variance formulas for both mbc and c-mbc. We compared the mbc and the c-mbc with commonly used concordance measures in a simulation study and in two external validation settings. The mbc was asymptotically equivalent to a previously proposed resampling-based case-mix corrected c-index. The c-mbc remained stable at the true value with increasing proportions of censoring, while Harrell's c-index and to a lesser extent Uno's concordance measure increased unfavorably. Variance estimates of mbc and c-mbc were well in agreement with the simulated empirical variances. We conclude that the mbc is an attractive closed-form measure that allows for a straightforward quantification of the expected change in a model's discriminative ability due to case-mix heterogeneity. The c-mbc also reflects regression coefficient validity and is a censoring-robust alternative for the c-index when the proportional hazards assumption holds. Copyright © 2016 John Wiley \& Sons, Ltd.","container-title":"Statistics in Medicine","DOI":"10.1002/sim.6997","ISSN":"1097-0258","issue":"23","journalAbbreviation":"Stat Med","language":"eng","note":"PMID: 27251001\backslash nPMCID: PMC5550798","page":"4136-4152","source":"PubMed","title":"A new concordance measure for risk prediction models in external validation settings","volume":"35","author":[\{"family":"Klaveren","given":"David","non-dropping-particle":"van"\},\{"family":"Gönen","given":"Mithat"\},\{"family":"Steyerberg","given":"Ewout W."\},\{"family":"Vergouwe","given":"Yvonne"\}],"issued":\{"date-parts":[["2016"]],"season":"15"\}\}\}],"schema":"https://github.com/citation-style-language/schema/raw/master/csl-citation.json"\} (2)$.

We have:

$$mROC(t)=1-\bar{F}_{1}\left( \bar{F}_{0}^{-1}\left( 1-t \right) \right),$$

where 0 ≤ 𝑡 ≤ 1 is the false positive probability, so

$$mAUC=\int_{0}^{1} \left\{ 1-\bar{F}_{1}\left( \bar{F}_{0}^{-1}\left( 1-t \right) \right) \right\}dt.$$

The change of variable $x=\bar{F}_{0}^{-1}\left( 1-t \right)$ leads to

$$mAUC=\int_{0}^{1} \left\{ 1-\bar{F}_{1}\left( x \right) \right\}\bar{f}_{0}\left( x \right)dx=1-P\left( \pi_{0}^{*}>\pi_{1}^{*} \right)=P\left( \pi_{1}^{*}>\pi_{0}^{*} \right)=mbc.$$

**Section 2:** Proof of the convergence of mROC and empirical ROC curves under model calibration

# **Lemma:** For a moderately calibrated risk prediction model, the empirical and model-based ROC curves asymptotically converge.

**Proof:** Let $\boldsymbol{X}$ be the vector of covariates (predictors), with $\mathbf{X}_{i}$ referring to the realization of this vector for the i^th^ individual. A pre-specified risk prediction model $\pi^{*}(\mathbf{X})$ yields predicted risks $\pi^{*}\equiv\pi^{*}(\mathbf{X}_{i})$. When sampling from a population, the mapping from $\mathbf{X}_{i}$to $\pi_{i}^{*}$ is known, but $\pi_{i}^{*}$ for the ith individual is random as $\mathbf{X}_{i}$ is randomly selected. For any value of the predicted risk $\pi^{*}$, there is a unique ‘calibrated risk’ $\pi$given by the true risk of the outcome among all individuals with that predicted risk: $\pi\equiv\pi(\pi^{*})=P\left( Y=1 | \pi^{*}\left( \mathbf{X} \right)=\pi^{*} \right).$ A model is moderately calibrated when $\forall z, \pi(z)=z$.

We first consider the behavior of $F_{1n}(t)$. For each fixed value of $t$, $F_{1n}(t)$ is the average of $I(\pi_{i}^{*}\leq t)$ among individuals with $Y_{i}=1$. Hence, provided $P(Y=1)>0$, dividing both the numerator and denominator of the expression for $F_{1n}(t)$ in the main text by $n$ and applying the Weak Law of Large Numbers (in what follows, an arrow denotes convergence in probability as the sample size *n* approaches infinity), yields:

$$F_{1n}\left( t \right)\to\frac{E[I\left( \pi^{*}\leq t \right).Y]}{E\left( Y \right)}=\frac{P\left( \pi^{*}\leq t, Y=1 \right)}{P\left( Y = 1 \right)}=P\left( \pi^{*}\leq t \right|Y=1)=F_{1}\left( t \right).$$

Bayes' rule allows this limit to be re-expressed as

$$P(\pi^{*}\leq t | Y=1)=\frac{P\left( Y=1 | \pi^{*}\leq t \right).P\left( \pi^{*}\leq t \right)}{P\left( Y = 1 \right)}.$$

Proceeding similarly for $\bar{F}_{1n}(t)$ leads to

$$\bar{F}_{1n}\left( t \right)\to\frac{E[I\left( \pi^{*}\leq t \right).\pi^{*}]}{E[\pi^{*}]}=\frac{P\left( \pi^{*}\leq t, Y^{*}=1 \right)}{P\left( Y^{*} = 1 \right)}=P\left( \pi^{*}\leq t | Y^{*}=1 \right)= \bar{F}_{1}\left( t \right).$$

Again, applying the Bayes’ rule, we have

$$P\left( \pi^{*}\leq t \right|Y^{*}=1)=\frac{P\left( Y^{*}=1 | \pi^{*}\leq t \right).P\left( \pi^{*}\leq t \right)}{P\left( Y^{*}= 1 \right)}.$$

For a moderately calibrated risk prediction model where ${\pi\left( \pi^{*} \right)=\pi}^{*} ,$it follows immediately that $P\left( Y = 1 \right)=E\left( \pi\right)=E\left( \pi^{*} \right)=P(Y^{*}=1)$. To prove $F_{1n}\left( t \right)-\bar{F}_{1n}\left( t \right)\to0$ we therefore only need to show that $P\left( Y=1 | \pi^{*}\leq t \right)-P\left( Y^{*}=1 | \pi^{*}\leq t \right)=0$. But we have

$P\left( Y=1 | \pi^{*}\leq t \right)-P\left( Y^{*}=1 | \pi^{*}\leq t \right)\propto\int_{0}^{t} \left\{ P\left( Y=1|\pi^{*}=z \right)-P\left( Y^{*}=1|\pi^{*}=z \right) \right\}.dP\left( \pi^{*}\leq z \right)=0$, by the definition of moderate calibration.

Similar arguments apply for $F_{0n}\left( t \right)$ and $\bar{F}_{0n}\left( t \right)$, thereby establishing the desired result.

**Section 3:** A stylized example demonstrating the connection between mROC and model calibration

Consider the simple situation when the true risk, represented by $p$, has a standard uniform distribution in the population:

$$p\sim uniform\left( 0,1 \right),$$

$$Y\sim Bernoulli\left( p \right).$$

We consider three scenarios: the ‘correct specification’ scenario, when the prediction model correctly estimates the true risk ($\pi^{*}=p$) and thus is calibrated, and two alternative scenarios of overestimation $(\pi^{*}=\sqrt{p})$ and underestimation ($\pi^{*}=p^{2}$) of the true risks. For these three scenarios, the analytical forms of the population-based CDFs $F_{1}\left( t \right)$, $F_{0}\left( t \right)$ , $\bar{F}_{1}(t)$, and $\bar{F}_{0}(t)$ are provided in ***Table S1***.

| ***Table S1***: Population-based forms of the cumulative distribution functions underlying the empirical and model-based ROC curves for the simple uniform risk situation | | | |
| --- | --- | --- | --- |
| **CDF** | **Scenarios** | | |
|  | Correct specification | Overestimated risk | Underestimated risk |
|  | $\pi^{*}=p$ | $\pi^{*}=\sqrt{p}$ | $\pi^{*}=p^{2}$ |
| $F_{1}(t)$ | $t^{2}$ | $t^{4}$ | $t$ |
| $F_{0}(t)$ | $2t - t^{2}$ | $2 t^{2} - t^{4}$ | $2 \sqrt{t} - t$ |
| $\bar{F}_{1}(t)$ | $t^{2}$ | $t^{3}$ | $t^{3/2}$ |
| $\bar{F}_{0}(t)$ | $2t - t^{2}$ | $3 t^{2} - 2 t^{3}$ | $(3\sqrt{t} - t^{\frac{3}{2}}) / 2$ |

For all three scenarios, given that the predicted risks are monotonically transformed versions of the true risk, the population-based ROCs are the same: $ROC\left( t \right)=2 \sqrt{t} - t$. However, $mROC(t)=ROC(t)$ only for the correct specification scenario. For the two alternative scenarios, closed-form expressions for $mROC(t)$ are not available, but the single root for ${\bar{F}_{0}}^{-1}(1-t)$ can be found numerically to evaluate $mROC(t)$. Results are provided in ***Figure S1***, where the ROC, mROC, and corresponding population-based calibration plots are provided for comparison. The latter have closed-form expressions in this simple situation, but in general calibration plots cannot be drawn without grouping or smoothing the data. On the other hand, the mROC curve can be evaluated from a sample without the requirement for any such arbitrary specifications.

| ***Figure S1:*** ROC and mROCs for the simple uniform risk situation (left) and the corresponding calibration plots (right). Black: fully calibrated model; red: over-estimated risk; blue: underestimated risk. For the left panel, the ROC curves coincide (black line) for the three scenarios considered, but the mROC curves are distinct. For the right panel, the calibration curves for the three scenarios are all distinct. | |
| --- | --- |
| 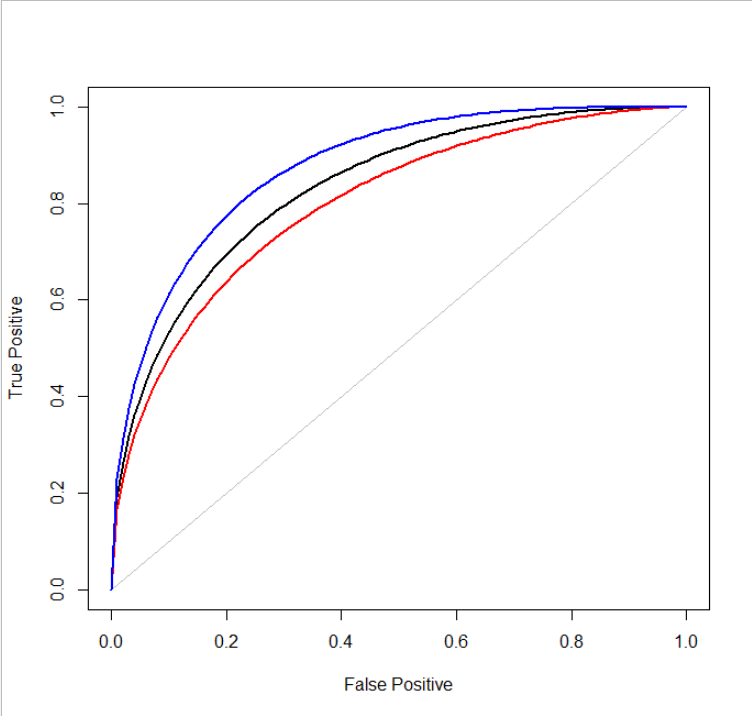 | 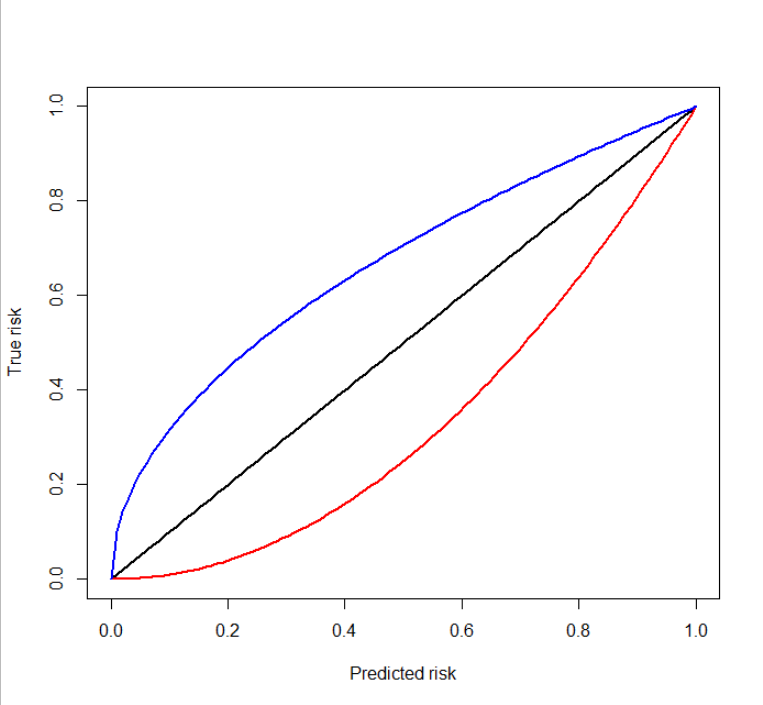 |

**Section 4:**  Sufficient conditions for moderate calibration

**Lemma:** If the expected value of the predicted and true risks are the same in the population, then pointwise equality of population ROC and mROC curves implies the model is at least moderately calibrated.

**Proof:** Let $\pi^{*}=\pi^{*}(\mathbf{X})$ represent the predicted risk, and $G^{*}(\cdot)$ its CDF. Let $\pi(\cdot)$ be the true calibration function, representing the mapping from $\pi^{*}$ to the actual risk: $\pi(z)=P\left( Y=1 \right|\pi^{*}=z)$. A model being at least moderately calibrated means $\pi(z)=z$ almost everywhere (a.e.) on the support of $G^{*}(\cdot)$.

Given that the result to be established is concerned with population quantities, in place of the CDFs $F_{1n}(t)$, $F_{0n}\left( t \right),$ $\bar{F}_{1n}\left( t \right)$, and $\bar{F}_{0n}\left( t \right)$ that underlie the empirical ROC and mROC curves, we use the limiting versions of these CDFs.

For the ROC curve, we can express the underlying CDFs as

$$F_{1}\left( t \right)=P\left( \pi^{*}\leq t | Y=1 \right)=\frac{P\left( \pi^{*}\leq t,Y=1 \right)}{P\left( Y=1 \right)}=\frac{E[I\left( \pi^{*}\leq t \right).\pi\left( \pi^{*} \right)]}{E[\pi\left( \pi^{*} \right)]}=\frac{\int_{0}^{t} \pi(u).dG^{*}(u)}{\int_{0}^{1} \pi\left( u \right).dG^{*}\left( u \right)},$$

and similarly,

$$F_{0}\left( t \right)=\frac{\int_{0}^{t} \left( 1-\pi\left( u \right) \right).dG^{*}\left( u \right)}{1-\int_{0}^{1} \pi\left( u \right).dG^{*}\left( u \right)}.$$

For the mROC curve, similar derivations result in

$$\bar{F}_{1}\left( t \right)=\frac{\int_{0}^{t} u.{dG}^{*}\left( u \right)}{\int_{0}^{1} u.{dG}^{*}\left( u \right)},$$

and

$$\bar{F}_{0}\left( t \right)=\frac{\int_{0}^{t} \left( 1-u \right).{dG}^{*}\left( u \right)}{1-\int_{0}^{1} u.{dG}^{*}\left( u \right)}.$$

For the sake of simplicity and to avoid technicalities around the behavior of the quantile function for discrete distributions, the proof presented here is for the common case where$G^{*}(\cdot)$ is a strictly increasing function without jumps (equivalently, it has a corresponding probability density function having no intervals with zero density). This is the case, for example, for typical logistic regression models when there is at least one continuous predictor with unrestricted range. Given this condition, $\bar{F}_{1}\left( t \right)$ and $\bar{F}_{0}\left( t \right)$ are strictly increasing (without jumps) on [0,1] and, with the additional technical condition that $0<\pi\left( z \right)<1$ (the true risk is not strictly 0 or 1 at any level of predicted risk), so too are $F_{1}(t)$ and $F_{0}\left( t \right)$.

With these expressions, we can re-express the result to be established as

$$\left\{ \begin{aligned} Condition 1: \int_{0}^{1} u.{dG}^{*}\left( u \right)=\int_{0}^{1} \pi\left( u \right).dG^{*}\left( u \right) \\ Condition 2: \forall t \bar{F}_{1}\left( {\bar{F}_{0}}^{-1}\left( 1-t \right) \right)=F_{1}\left( {F_{0}}^{-1}\left( 1-t \right) \right) \end{aligned} \right. \Longrightarrow\pi\left( z \right)=z a.e.$$

Let $a=a(t)={\bar{F}_{0}}^{-1}(1-t)$ and $=b(t)={F_{0}}^{-1}\left( 1-t \right)$; it follows that $\bar{F}_{0}\left( a \right)=F_{0}\left( b \right)$. Then *Condition 2***,** and the strictly increasing nature of the CDFs, imply:

$$\bar{F}_{0}\left( a \right)=F_{0}\left( b \right)\Leftrightarrow\bar{F}_{1}\left( a \right)=F_{1}\left( b \right).$$

The expressions above for these CDFs yield the equivalent statement (after making use of *Condition 1*) that, for each fixed $t$:

$$\int_{0}^{a} \left( 1-u \right).{dG}^{*}\left( u \right)=\int_{0}^{b} [1-\pi(u)].{dG}^{*}\left( u \right)\Leftrightarrow\int_{0}^{a} u.dG^{*}\left( u \right)=\int_{0}^{b} \pi\left( u \right).dG^{*}\left( u \right),$$

or equivalently,

$$\int_{0}^{a} u.{dG}^{*}\left( u \right)=\int_{0}^{b} \pi\left( u \right).{dG}^{*}\left( u \right)\Leftrightarrow G^{*}\left( a \right)=G^{*}\left( b \right).$$

Let ${G^{*}}^{-1}(.)$ be the quantile function of $G^{*}\left( . \right)$. Setting $x=x\left( t \right)=G^{*}\left( a \right)=G^{*}\left( b \right)$, the previous statement can be written as:

$$\forall x \int_{0}^{{G^{*}}^{-1}\left( x \right)} u.{dG}^{*}\left( u \right)=\int_{0}^{{G^{*}}^{-1}\left( x \right)} \pi\left( u \right).dG^{*}\left( u \right).$$

With a change of variable $y=G^{*}\left( u \right)$, this becomes:

$$\forall x \int_{0}^{x} {G^{*}}^{-1}\left( y \right).dy=\int_{0}^{x} \pi\left( {G^{*}}^{-1}\left( y \right) \right).dy,$$

implying that $\pi(z)=z$ almost everywhere on the support of $G^{*}(\cdot)$, the probability distribution of the predicted risks.

**Section 5:** Calculating a unified p-value for the assessment of model calibration

1. Calculate $A_{n}$ and $B_{n}$ from the vectors of $\boldsymbol{\pi}^{\mathbf{*}}$ and $\mathbf{Y}$. These are the point estimates of the test statistics.
2. For i=1 to N (number of simulations):

2.1. Generate a random response vector $\mathbf{Y}_{i}^{*}$ from the predicted risks $\boldsymbol{\pi}^{\mathbf{*}}$.

2.2. Calculate $A_{0i}$ and $B_{0i}$from $\boldsymbol{\pi}^{\mathbf{*}}$and $\mathbf{Y}_{i}^{*}$and store their values.

1. Based on the $A_{0i}s$ and $B_{0i}s$, construct the empirical CDFs $eCDF_{A_{n}}\left( . \right)$ and $eCDF_{B_{n}}\left( . \right)$.
2. Calculate $p_{A_{n}}=1-eCDF_{A_{n}}(A_{n})$, $p_{B_{n}}=1-eCDF_{B_{n}}(B_{n})$, and $U_{n}=-2.\left[ \log\left( p_{A_{n}} \right)+\log\left( p_{B_{n}} \right) \right]$.
3. For each simulated vector $\mathbf{Y}_{i}^{\mathbf{*}}$, use the same empirical CDFs to calculate simulated p-values $p_{A_{i}}$, $p_{B_{i}}$, and test statistic $U_{n_{i}}$. For these N values of $U_{n_{i}}$, calculate $c=\frac{var\left( U_{n} \right)}{2.average\left( U_{n} \right)}$ and $k=\frac{2.average\left( U_{n} \right)^{2}}{var\left( U_{n} \right)}$.
4. The unified p-value is evaluated as $p_{U_{n}}=1-F\left( \frac{U_{n}}{c};k \right),$ where $F\left( .;k \right)$ is the CDF of the chi-square distribution with $k$ degrees of freedom.

**Section 6:** Methods and results of the first set of simulations

We generated a single predictor $X\sim Normal(0,1)$, and modeled the true risk as $p=1/(1+\exp\left( -X \right))$, resulting in the population average response probability of 0.5. We then evaluated the performance of the test in a simulated independent sample of $n$ observations when the predicted risks suffer from various degrees of mis-calibration. This was modeled by applying a logit-linear transformation of the true risks to generate the predicted risks: $logit\left( \pi^{*} \right)=a+b.X$. We simulated response values and predicted risks under a fully factorial design with values $a=\left\{ -0.25, -0.125, 0, 0.125, 0.25 \right\}, b=\left\{ 0.5, 0.75, 1, 1.5, 2 \right\},$ creating 25 simulation scenarios each for $n=\{100, 250, 1000\}$.

In this particular setup, if $Y$ is the observed binary response, a likelihood ratio test for $\beta_{0}=0$ and $\beta_{1}=1$ in the logistic model$logit(P(Y=1))=\beta_{0}+\beta_{1}.logit(\pi^{*})$, can be used to test for moderate calibration. This model is equivalent to a typical logistic regression model where the logit-transformed predicted probabilities are considered a covariate. In general, the likelihood ratio test is a test for ‘weak calibration’ in the hierarchical definition of model calibration proposed by Van Calster et al, with weak calibration achieved if $\beta_{0}=0$ and $\beta_{1}=1$(1). However, in this setup, it is a valid test for moderate calibration because the link function (logit) is known and the associations are known to be linear on the logit scale (therefore weak and moderate calibration are equivalent in this setting). As such, and according to the Neyman-Pearson lemma, the likelihood ratio test is the most powerful test for moderate calibration in this setup, providing a yardstick to evaluate the performance of the proposed test.

The relationships between the predicted and true risks are depicted in ***Figure S2.*** The ROC and mROC curves are presented in ***Figure S3***. Results of the simulation studies, in terms of the proportion of times the null hypotheses were rejected, are provided in ***Figure S4.*** As these results demonstrate, the unified test performs very similarly to the likelihood ratio test.

| ***Figure S2:*** Relationship between predicted (X axis) and true (Y axis) risks. |
| --- |
| 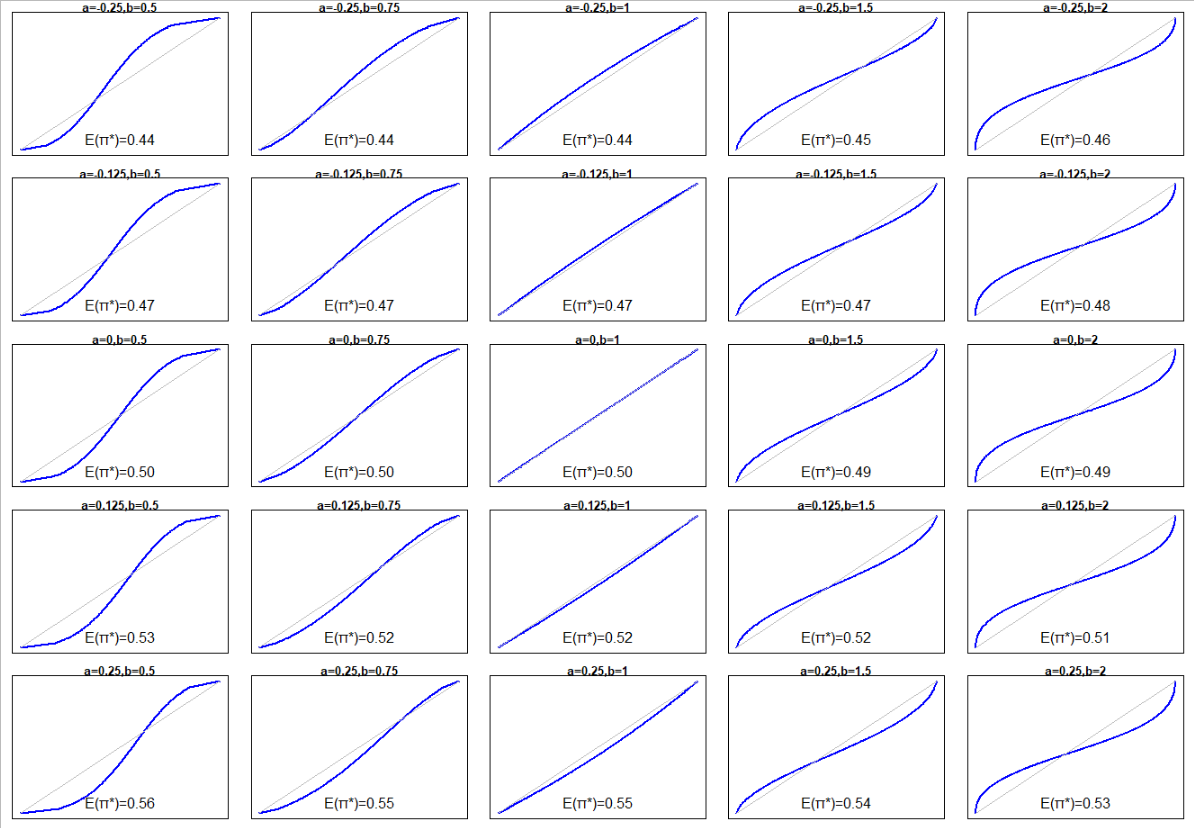 |

| ***Figure S3:*** ROC (black) and mROC (red) curves for the simulation scenarios. The panels positionally correspond to the calibration plots and simulation parameters presented in Figure S2. |
| --- |
| 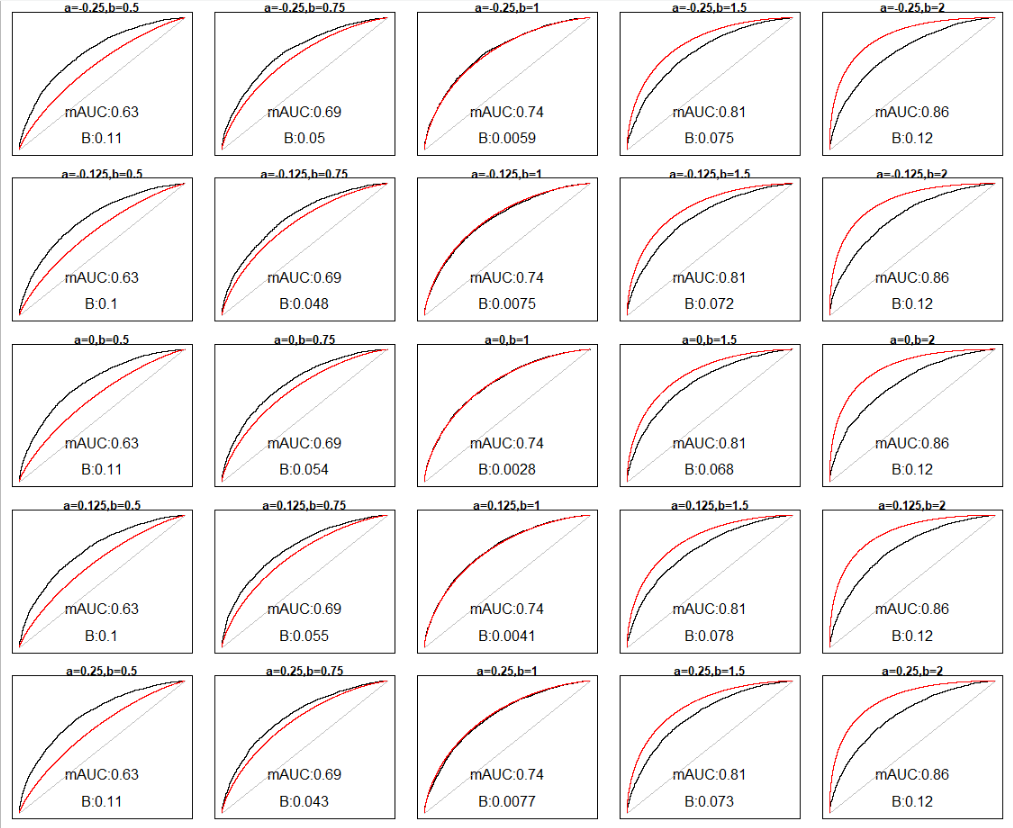 |

| ***Figure S4:*** Probability of rejecting the null hypothesis for the mean calibration (white bar), ROC equality (gray bar), unified (orange bar), Hosmer-Lemeshow (dark blue bar), and Likelihood Ratio (light blue bar) tests. The panels positionally correspond to the calibration plots and simulation parameters presented in Figure 2. |
| --- |
| 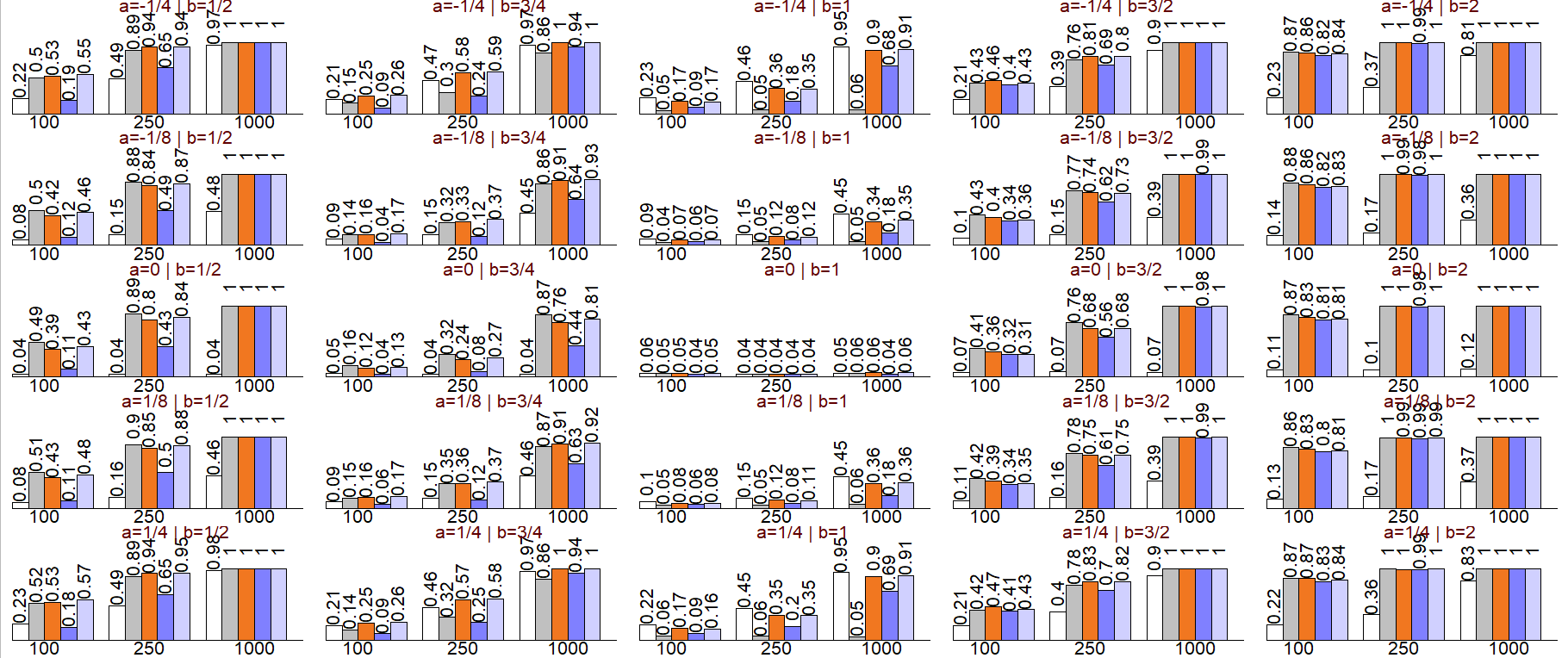 |

**References**

1. Van Calster B, Nieboer D, Vergouwe Y, De Cock B, Pencina MJ, Steyerberg EW. A calibration hierarchy for risk models was defined: from utopia to empirical data. J Clin Epidemiol. 2016;74:167–76.

2. van Klaveren D, Gönen M, Steyerberg EW, Vergouwe Y. A new concordance measure for risk prediction models in external validation settings. Stat Med. 2016 15;35(23):4136–52.
